# Supplementary material for: Intraocular Injection of ES Cell-Derived Neural Progenitors Improve Visual Function in Retinal Ganglion Cell-Depleted Mouse Models
Source: Front Cell Neurosci. 2017 Sep 20;11:295. doi: 10.3389/fncel.2017.00295 (PMC5611488; doi:10.3389/fncel.2017.00295)
Supplement: Supplementary file 1 [file Presentation_1.pdf]

Supplementary Information

Title: Intraocular injection of ES cell-derived neural progenitors improve visual function in retinal ganglion cell-depleted mouse models

Mundackal Sivaraman Divya[1,3], Vazhanthodi Abdul Rasheed[1,4], Tiffany Schmidt[2,5], Soundararajan Lalitha[1], SamerHattar[2] and Jackson James [1*]

[1] Neuro-Stem Cell Biology Laboratory, Neurobiology Division, Rajiv Gandhi Centre for Biotechnology, Thiruvananthapuram, Kerala-695 014, India

[2] Department of Biology, Johns Hopkins University, Baltimore, MD-21210, USA

[3] Present address: Cell Conversion Technology Unit, RIKEN Center for Life Science Technologies, Yokohama-2300045, Japan

[4] Present address: Department of Zoology, Government College Chittur, Palakkad, Kerala-678104, India

[5] Present address: Department of Neurobiology, Northwestern University, Evanston, IL 60053, USA

Fig. S1

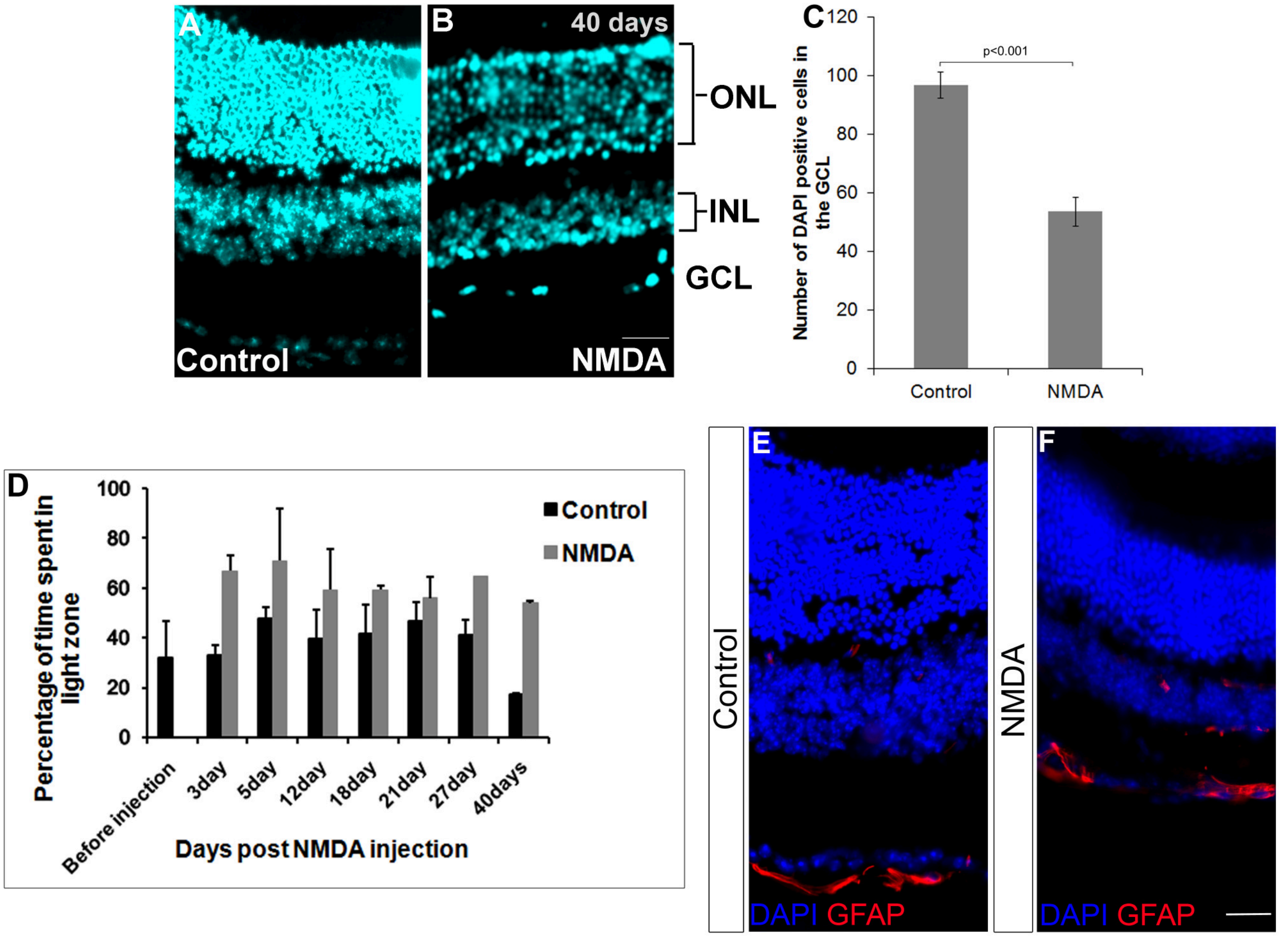
A
B
40 days
ONL
INL
GCL
Control
NMDA
C
Number of DAPI positive cells in the GCL
p<0.001
0
20
40
60
80
100
120
Control
NMDA
D
Percentage of time spent in light zone
Control
NMDA
Before injection
3day
5day
12day
18day
21day
27day
40days
Days post NMDA injection
E
F
Control
NMDA
DAPI GFAP
DAPI GFAP

Fig. S2

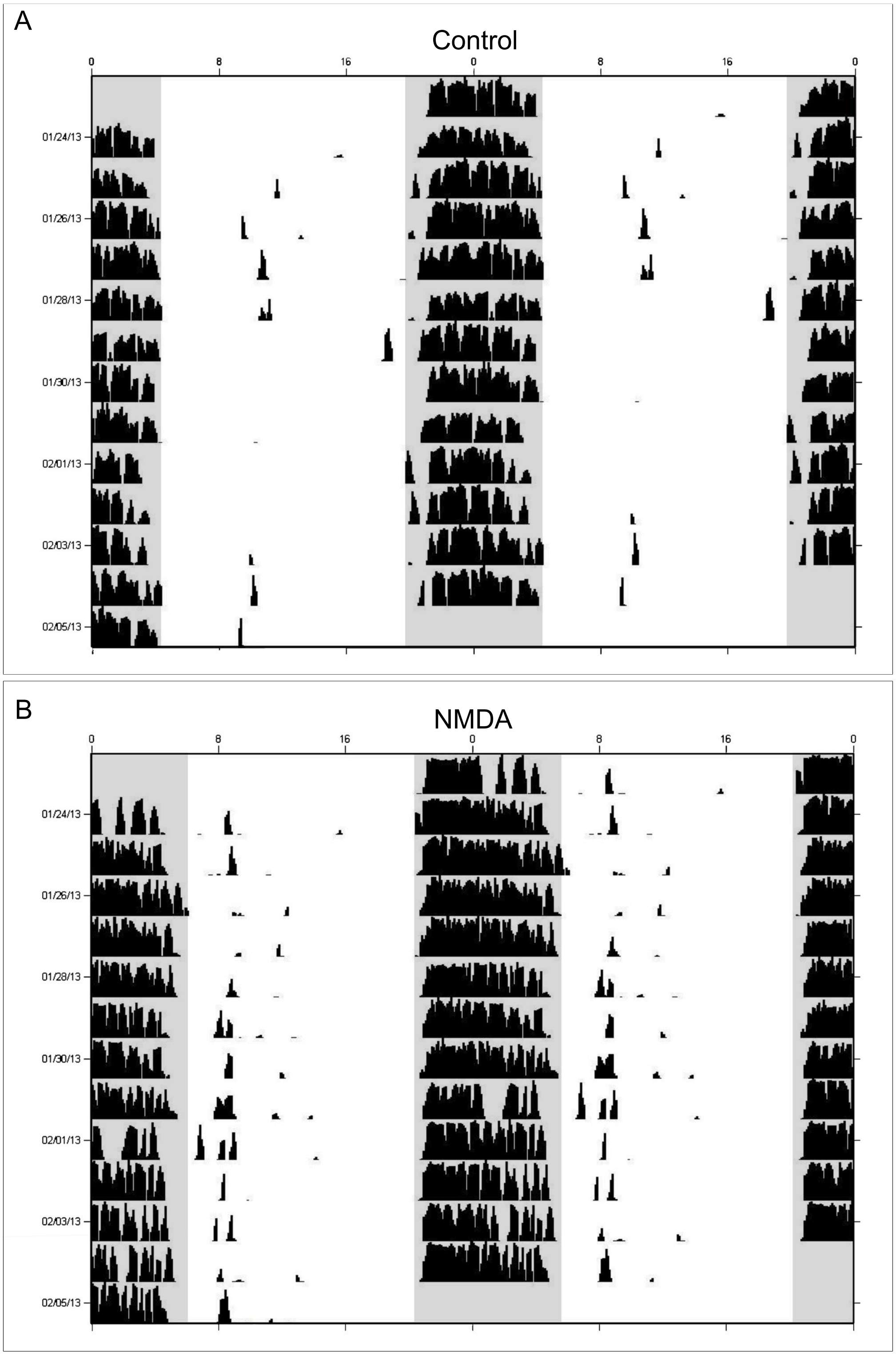
A
Control
0
8
16
0
8
16
0
01/24/13
01/26/13
01/28/13
01/30/13
02/01/13
02/03/13
02/05/13
B
NMDA
0
8
16
0
8
16
0
01/24/13
01/26/13
01/28/13
01/30/13
02/01/13
02/03/13
02/05/13

Fig. S3

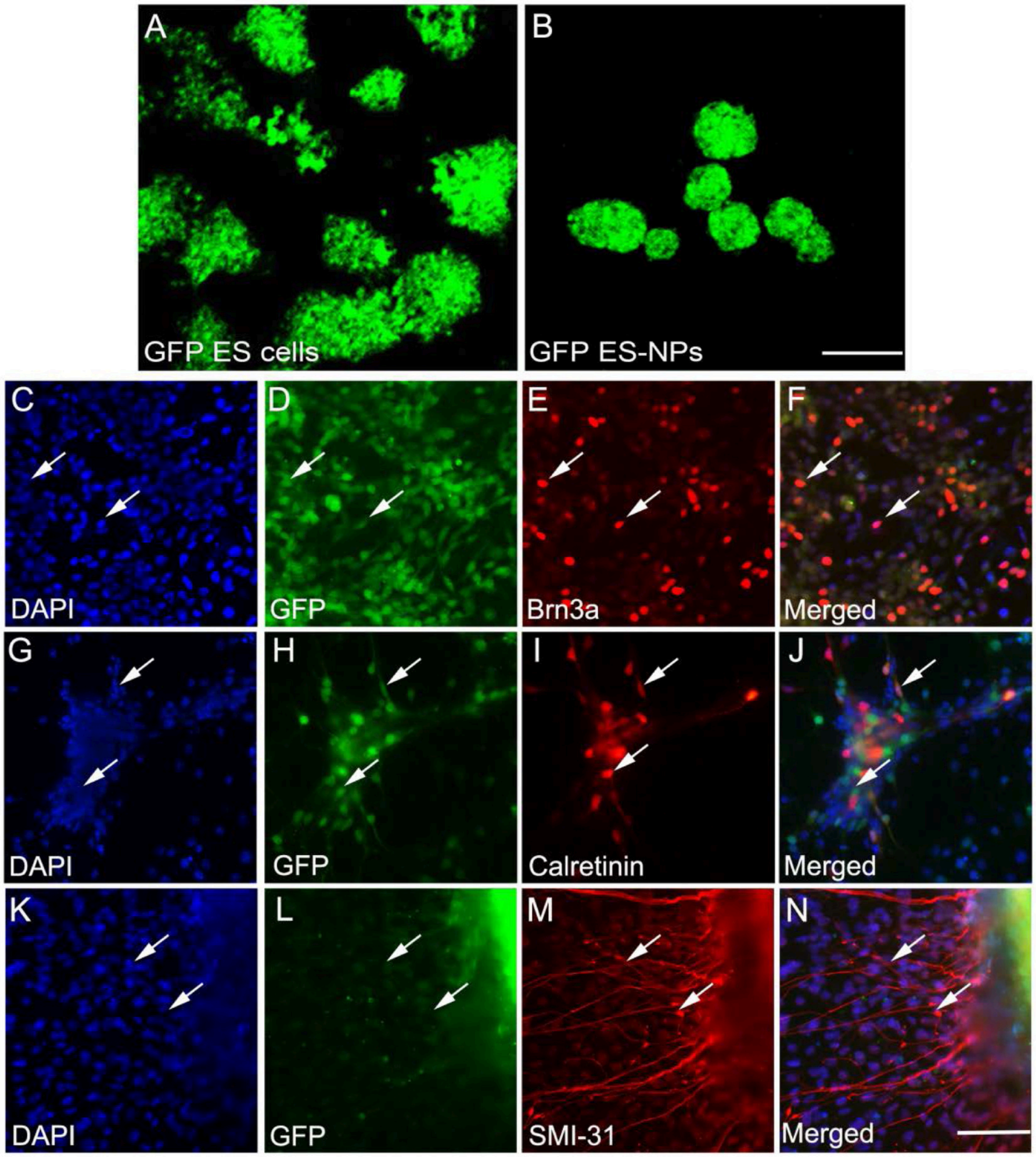
A
GFP ES cells
B
GFP ES-NPs
C
DAPI
D
GFP
E
Brn3a
F
Merged
G
DAPI
H
GFP
I
Calretinin
J
Merged
K
DAPI
L
GFP
M
SMI-31
N
Merged

Supplementary figure legend

Figure S1. Degeneration of RGC layer after 40 days post NMDA injection. NMDA effectively reduced the thickness of retinal layers with significant loss of RGCs in NMDA injected group (B) than compared to controls (PBS injected group) (A). (C) Graph represents the number of DAPI positive cells in the GCL layer of control and NMDA injected animals, indicating ~50% reduction of cells in the GCL. (D) Graph represents the percentage of time spent by the control and NMDA injected animals in the light chamber. NMDA injected animals spent more time in the light chamber than the controls as obtained from light avoidance behavioural experiments. (E & F) GFAP immunostaining in the control and NMDA injected retina. Data are expressed as Mean ± SD from triplicates of three different experiments. Number of animals used, Control = 6, NMDA injected = 6. Scale = 50µm.

Figure S2: Non image forming functions are not affected in NMDA-injected mouse models. (A) Wheel running activity demonstrated in the form of actograms showing normal circadian rhythm in control wild type animals. (B) NMDA-injected animals also showed good wheel running activity and thus a normal circadian rhythm similar to that of control animals.

Figure S3: GFP-expressing ES cell line (CE3 ES cells, ATCC SCRC-1039) and itsdifferentiation into RGC lineage. Stable GFP-expressing ES cells (A) and ES-NPs generated from those ES cells (B). GFP-expressing ES-NPs differentiated into RGC-like cells as evidenced by the co-expression of RGC markers Brn3a, Calretinin and SMI-31 with GFP (C-N). Scale =50µm.
